# The significance of Bartonella henselae bacterias for oncological diagnosis in children

**DOI:** 10.1186/s13027-015-0025-x

**Published:** 2015-09-14

**Authors:** Katarzyna Mazur-Melewska, Katarzyna Jończyk-Potoczna, Anna Mania, Paweł Kemnitz, Jarosław Szydłowski, Wojciech Służewski, Magdalena Figlerowicz

**Affiliations:** Department of Infectious Diseases and Child Neurology Karol Marcinkowski University of Medical Sciences, Szpitalna Street 27/33, 60-578 Poznań, Poland; Pediatric Radiology Department Chair of Radiology Karol Marcinkowski University of Medical Sciences, Szpitalna Street 27/33, 60-578 Poznań, Poland; Pediatric ENT Department, Karol Marcinkowski Poznan University of Medical Sciences, Szpitalna Street 27/33, 60-578 Poznań, Poland

**Keywords:** Bartonella henselae, CSD, Children

## Abstract

**Background:**

Cat-scratch disease (CSD) is a common infection in children; however, the wide spectrum of its clinical picture may lead to delayed diagnosis. An unusual presentation of CSD includes in the differential diagnosis malignant diseases, Epstein-Barr and cytomegalovirus infections, tuberculosis, and mycobacterioses. The diagnostic procedure is difficult, and it is important to consider CSD as the etiology of untypical lesion.

**Patients and method:**

We present the analysis of 22 immunocompetent children treated with the clinical diagnosis of CSD in our hospital. Their ages were 2 to 16 years (mean 9.15 ± 2.2 years). Four of them presented classical papulas at admission time. Asymmetric, local lymphadenopathy was present in 16 patients. Five children, who presented an untypical course of CSD mimicking the oncological process, were analysed carefully. There were 3 patients with skull osteomyelitis, 1 with inflammation of the parotid gland, and 1 with an extra peripharyngeal mass. The diagnosis in these children was based on epidemiological, radiological, serological, and histological factors.

**Results:**

About 25 % of children with bartonellosis present an untypical spectrum of symptoms, including the lack of documented cat contact, primary lesions, or peripheral lymphadenopathy. Radiological methods like USG, CT, MRI present the unspecific masses, but they are not enough to distinguish the *Bartonella* inflammatory and oncological process. The final diagnosis was based on a histological method with additional polymerase chain reaction test.

**Conclusion:**

CSD should be considered in differential diagnosis of any patient with untypical lesions located on the head, neck, and upper extremities.

*Bartonella* species are small Gram-negative bacteria which have been isolated from humans and mammals. The reservoirs of *Bartonella henselae* are domestic animals: cats, guinea pigs, rabbits, and occasionally dogs [1]. The cat flea *Ctenocephalides felis* (*Siphonaptera: Pulicidae*) is the most well-recognised vector of *B. henselae*, and transmission between cats and humans mainly occurs through infected flea faeces. New potential vectors are confirmed to be capable of transmitting bartonellosis, in particular *Ixodes ricinus*, the most widespread ixodid tick in Western Europe, which is frequently associated with bites in humans [2–4].

The wide spectrum of diseases caused by these bacteria range from asymptomatic cases to skin lesions in the inoculation place, fever of unknown origin, local lymphadenopathy, hepatomegaly, ostemyelitis, encephalopathy, and endocarditis. In immunocompromised patients, *Bartonella sp.* can cause opportunistic infections like bacillary angiomatosis and peliosis hepatitis [5].

People become infected by being bitten or scratched by an infected animal [1, 6]. The disease begins with an erythematous papule (single or in a group) at the site of inoculation. Diagnosis is more probable if the doctor has all information about cat scratching in the patient’s history or finds visible signs of a cat’s aggression [5]. The papule appears 3 to 10 days after inoculation. The lesions progress through erythematous, vesicular, papular, and crusted stages. In classic cat-scratch disease (CSD), regional lymphadenopathy occurs 1 to 3 weeks after inoculation and lasts for up to several months. Eighty-five percent of infected people have a single group of nodes involved. Most frequently, the lymphadenopathy occurs in the axillary and epitrochlear nodes (46 %), head and neck (26 %), and the groin (17.5 %). The lymph nodes are painful, but movable with solid consistency. About 50 % of patients present CSD with systemic symptoms like generalised aches, malaise, anorexia, nausea, and abdominal pain [8].

About 10 % of patients present perinodal forms of bartonellosis, which are manifested by endocarditis, encephalitis, uveitis, conjunctivitis, hepatitis, and tuberous sclerosis. Incidentally, inflammation in the musculoskeletal system, like osteitis, artropathy, and myalgia, has also been described [7].

The aim of our study was to present untypical courses of CSD in children on the background of all diagnosed cases and to point out the diagnostic difficulties in differentiation between the inflammatory and oncological process.

## Material

This retrospective analysis of the medical documentation was performed. The patients were hospitalised in the Department of Infectious Diseases and Child Neurology at the University of Medical Sciences in Poznań and consulted in an outpatient clinic of infectious diseases between 2009 and 2014. Data were collected from 22 patients aged 2 to 16 years (mean 9.15 ± 2.2 years).

The subjects included 14 boys and 8 girls (Table 1).

**Table 1:** Characteristics of children with diagnosed *Bartonella henselae* infection

Number of children		22	
Age (years)		4–16 years (mean 9.15 ± 2.2)	
Contact with cats		14	
Symptoms:	Classical papula	4	
	Local lymphadenopathy	16	
	Prolonged fever	1	
	Tumour in internal organs	5	Parotide tumour – 1;
			Bone tumor – 3;
			Lymphoma-like mass in the neck area – 1

## Methods

### Radiology

Routine ultrasonography (USG) of the abdomen and lymph nodes of the neck was performed with the child in the supine or the right anterior oblique position. All USG examinations were performed with commercially available high-end ultrasound units (Philips iU22), a 7-to-12 MHz linear probe, and a 4–9 MHz convex probe. No patient underwent color Doppler or contrast-enhanced USG examination.

Two children whose ultrasound imaging revealed lesions in the organs underwent a computer tomography (CT) scan as an additional examination. Conventional CT was performed using a 128-layer Somatom Definition AS apparatus (Siemens). The intravenous contrast, 1–1.5 ml /kg (according to the patient’s age), was given with a flow rate of 1–2 ml/sec. The CT investigations were reviewed by two independent radiologists who reached a consensus interpretation. Both radiologists had the information about patients’ clinical status, but not about the diagnosis.

Magnetic resonance imagination (MRI) was performed in three children between the 4^th^ and 8th day of admission using the 3-Tesla scanner (MAGNETOM Spectra, Siemens Healthcare, Erlangen/Germany). T1-weighted examination was performed before and after intravenous administration of gadobutrol (Bayer Vital, Leverkusen/Germany, 0.1 mmol/kg body weights).

### Histology

The excisional biopsy was performed in 8 patients: 3 with peripheral lymph nodes enlargment and 5 children with perilymphatic localisation of the disease. All samples were fixed in formalin and processed for histologic analysis in hospital reference center. Stains used included Gram, hematoxylin and eosin, periodic acid–Schiff and Ziehl-Neelsen.

### Serology

Serum samples were tested for the presence of *B. henselae* specific antibodies using commercially available, indirect, immunofluorescence test (MRL Diagnostic, USA). For the MRL kit, immunoglobulin G (IgG) and IgM were tested separately. According the producer refererences the dilutions: 1:20 for IgM and 1:64 for IgG were regarded as the positive. The analysis was performed in the Unit of Rickettsiae, Chlamydiae, and Spirochaetes in the National Institute of Public Health in Warsaw.

### PCR

DNA was extracted from the material extracted during biopsy. Extracted DNA was subjected to polymerase chain reaction (PCR)-mediated amplification of the 16S-23S rRNA fragment gene characteristic for *Bartonella* species. The test was performed in the Unit of Rickettsiae, Chlamydiae, and Spirochaetes in the National Institute of Public Health in Warsaw. The result was described as positive or negative detection and differentiation of *Bartonella* species material.

This retrospective study was approved by the Local Human Investigation Review Board and the Ethics Committee of the University of Medical Sciences in Poznań, Poland. The radiological investigations were part of the routine diagnostic procedure. All the procedures were documented in our patient record files.

## Results

**Fig. 1:**
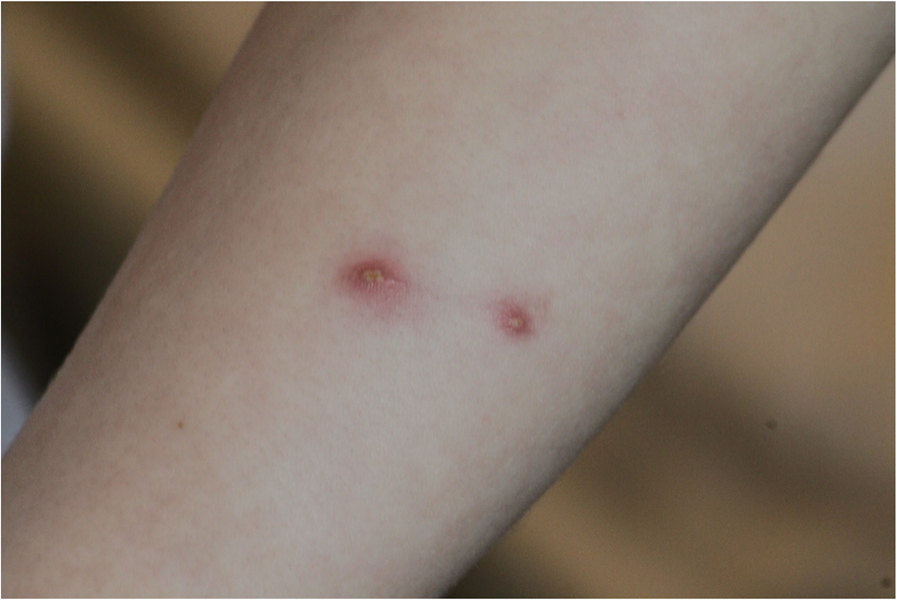
Cat scratch disease – active skin lesions (one week after inoculation)

**Fig. 2:**
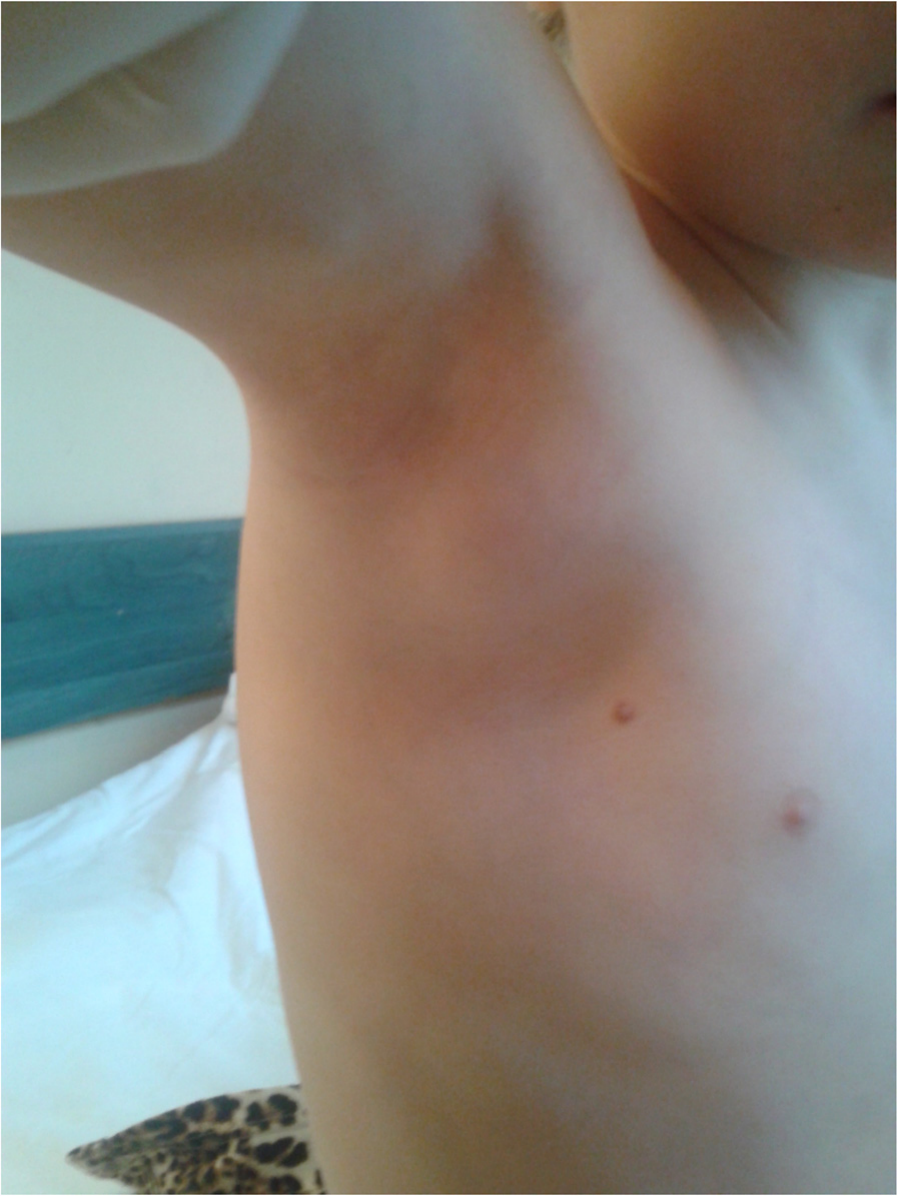
Peripheral, axillary lymphadenopathy in 5-years old child with CSD

**Fig. 3:**
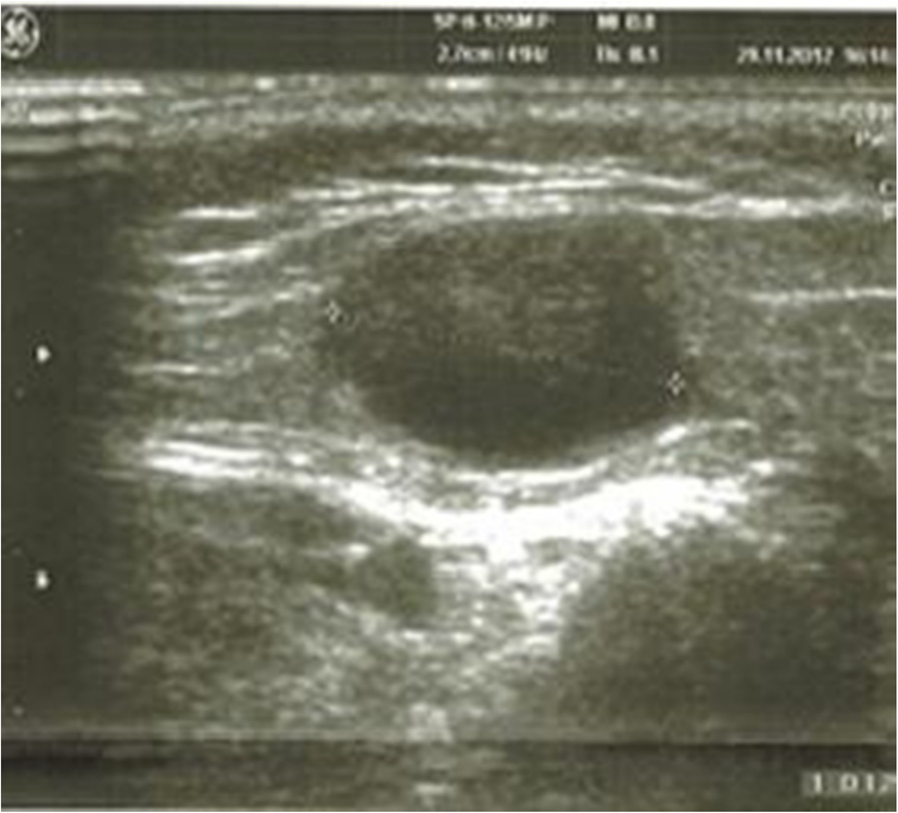
The ultrasound picture of hypoechoic lymph node in the 5-years-old child with CSD

All analysed children with CSD were immunocompetent. Their age was 2 to 16 years (mean 9.15 ± 2.2 years) without special predilection. Only 4 (18 %) children presented classic papules at admission time (Fig. 1). Lymphadenopathy was present in 16 (72 %) patients, primarily in the axillary nodes, in frequency followed by the cervical and inguinal areas (Figs. 2, 3). The nodes were often painful but the skin on them was not inflammated (Table 1).

One boy had a history of prolonged fever and fatigue that lasted about one month. He lived in the forest with his family, and five years ago he had been treated because of Lyme disease. He was bitten by ticks many times after. The serological test for *B. henselae* made from the blood was positive with ratio 1:256. The therapy with gentamycin (5 mg/kg/day) was effective within 72 h; he continued treatment for four weeks.

Five children who presented an untypical course of CSD mimicking the oncological process were analysed carefully. The clinical characteristics of these patients are shown in Table 2. All of them presented very high fever and local or generalised lymphadenopathy. The lymph nodes were solid, not movable, and connected with subcutaneous tissue. Two patients developed following symptoms; even their lymphadenopathy was treated with success. Most common in our observation was the osteomyelitis of the skull bones, which was found in 3 patients. One patient presented the enlargement of parietal gland and 1 the extra lesion in the peripharyngeal area. All of these 5 children had the radiological imaginations performed, but none of them suggested inflammation. The radiological imaginations (USG, CT, MRI) presented untypical lesions which mimicked the neoplastic process. Therefore, excisional biopsies of the masses and histopathology were performed. All diagnoses were based on the histopathological description. In four cases, the PCR from bioptical material was performed.

**Table 2:** Clinical characteristics of 5 patients presented atypical form of *B. henselae* infection

Age (years)/Sex	Clinical Features	Contact with cat	Radiological imagination	ELISA test (IgG/ratio)	The PCR made from the exceed material	Treatment
6/M	Enlargement of parotid gland in the left side. The gland was not painful but hard with the diameter 3 cm	scratched in the left eyelid	USG: solid, hypoechogenic lesion inside the parotid gland suggested neoplastic lesion. (Fot.3)	1: 512	positive	Claritromycin, surgical removing, trimetoprim/sulfametoxazol
2/M	Fever, arthralgia, generalized lymphadenopathy, left submandibular tissue inflammation, disseminated, tuberous lesions on the skull.	Cat at home	MRI imagination: the disseminated, pathological infiltration of the both parietal and left frontal bones with osteollysis, settled into the skull with not interrupted pachymeninx. The radiological suggestion: histicytosis.	1:256	-	Clindamycin, clarytromycine, ceftazydym), surgical biopsy
			USG: left submandibular lymphadenopathy sized 20 × 15 mm.			
7/M	Fever, local, cervical lymphadenopathy. The painful tumor on the occipito-parietal left area on the skull.	No	CT imagination: the local, pathological infiltration of the bones in the ocipitoparietal border without pachmeninx interruption	1:512	positive	Clindamycin, clarytromycin, surgical biopsy
12/F	The local, right axillary and ulnary lymphadenopathy with high fever. After 5–6 weeks the painful tumor on the right parietal area appeared.	scratched in the right hand	MRI imagination: the local, pathological infiltration of the parietal bone, settled into the skull with not interrupted pachymeninx. The radiological suggestion: histicytosis (Fig. 4).	1:1024	positive	Claritromycin, surgical biopsy, azitromycin, trimetoprim/sulfametoxazol
5/M	Severe headache, fever and torticollis. The physical examination presented the limitation of the head movements, mild enlargement of the left cervical lymph nodes	No	USG: left submandibular and cervical lymphadenopathy and hypoechogenic lesion sized 21 × 22 mm located medially to the big neck vessels.	1:512	positive	Ceftriakson, surgical biopsy
			MRI: the solid mass sized 20 × 14 × 30 mm in the peripharyngeal area modulated the left neck vessels and left tonsils. The lesion presented diffusion restriction in DWI MRI (Fig. 5)

**Fig. 4:**
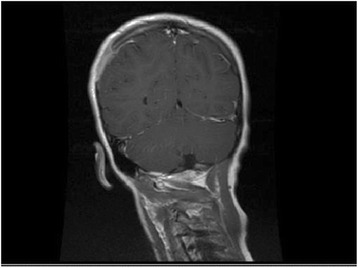
The cranial CT scan presented the solitary tissue mass overlying a skull lesion suggesting histiocytosis X in 12-years-old girl

**Fig. 5:**
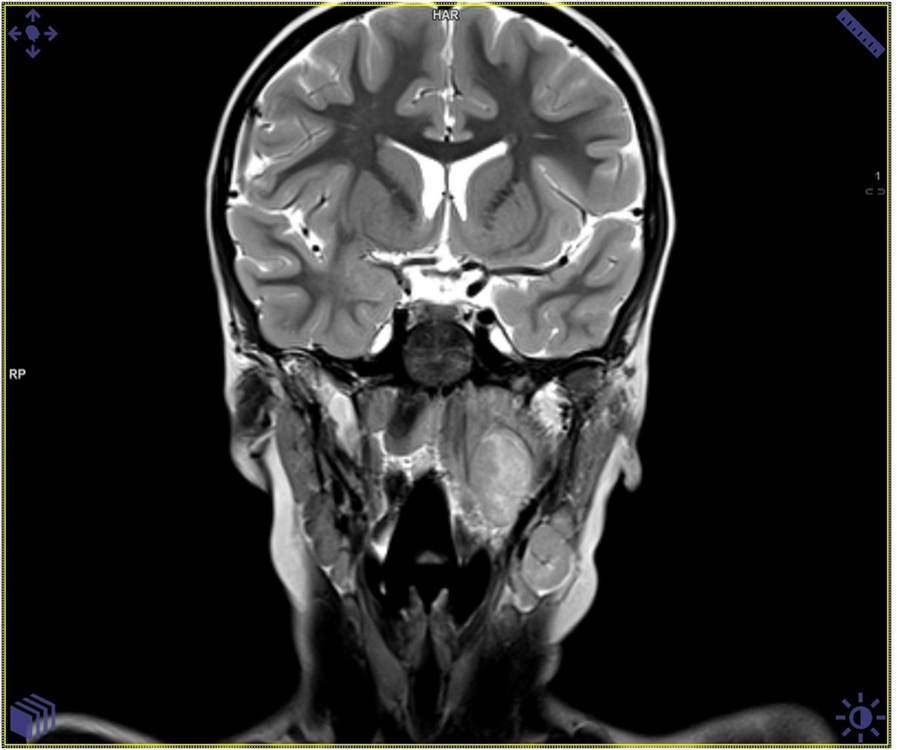
MRI scan of the head and neck of 5-years old child presented the solid mass in the peripharyngeal area.  Sized 20 × 14 × 30 mm lesion modulatesd the left neck vessels and left tonsil

Serologic tests for detection of antibodies to *B. henselae* in blood and PCR from resected material effectively provided laboratory confirmation of the diagnosis.

## Discussion

CSD is one of the conditions that causes nonspecific signs and symptoms. The typical presentation of a papula on the skin scratched by a cat is rather rare in clinical practice. CSD usually seems to be a relatively benign and self-limited disease which resolves without antimicrobial therapy. Probably many patients don’t pay attention to their small skin lesion and they avoid the doctor’s consultation. This is the reason that the most common clinical presentation of bartonellosis is local lymphadenopathy. A differential diagnosis of lymphadenopathy associated with CSD may include an oncological process (soft tissue sarcoma, soft tissue metastasis, and soft tissue lymphoma) and infectious lesions like Castlemann disease. There are a lot of descriptions presenting CSD as a skin cancer, lymphomas, Hodgkin disease and Kaposi sarcomas [8]. The unusual presentations suggest also that the infection can mimicate: a neuroblastoma, liver neoplasms and a breast cancer [9, 10]. The differencial procedure is especially difficult because the systemic infectious disease can mask the oncological process, and the positive ELISA test made from the blood can be the reason for a medical mistake, as it had been presented in our works [11].

The most frequently used tests for CSD diagnosis are serologic methods: indirect fluorescence assay (IFA) and enzyme immunoassay (EIA). The duration of serologic detection of antibodies is important for determining acute infection versus historical exposure. Positive immunoglobulins M (IgM) indicate acute disease, but their duration in the blood is approximately 100 days after exposure. This short duration means that IgM are identified only in 50 % of infected individuals. Immunoglobulins G (IgG) against *Bartonella henselae* are detectable up to 22–28 weeks after inoculation. As 25 % of patients remain seropositive for IgG after 1 year, it is difficult to diagnose active infection compared with previous infection [7, 12]. Additional disadvantages to serological tests include variable specificity and sensitivity, inability to differentiate between active and prior infection, and lack of *Bartonella*-specific antibody reaction, resulting in crossreactivity.

The most advanced technique involves the detection of bacterial material in patient’s tissues. There have been 3 main approaches to using PCR to diagnose *Bartonella* infection: amplification of the 16S rRNA gene, amplification of the citrase synthase gene (*gltA*), and amplification of the *htrA* gene of *B. henselae*. The specificity of PCR is nearly 100 %, but sensitivity ranges from 43 % to 76 %. In fact, the detection of *Bartonella sp*. in clinical material is equivalent to the level of isolation in culture [13, 14].

The radiological imaginations can be potential techniques for differentiation between benign and malignant lymph nodes [5]. In classic CSD, ultrasonography presents the enlarged lymph nodes, usually with central necrosis, associated with surrounding edema in the area of lymphatic drainage proximally to the site of inoculation [15]. CT shows lymphadenitis with central necrotic nodes. MRI imaging shows regional infectious lymphadenopathy as heterogeneous masses with surrounding edema characterised by a hypointense mass on T1-weighted images and hyperintense mass on T2-weighted images with peripheral enhancement after intravenous gadolinium administration. T2-weighted MRI shows surrounding edema more clearly than a CT scan [16]. Although imaging findings usually show nonspecific lymphadenitis, the histopathological examination connected with the PCR made from the material removed seems to be necessary to prove *B. henselae* as the cause of disease. Histological examination usually shows epitheloid granulomatous inflammation with multinucleated giant cells and central necrosis. PCR of the biopsy specimen is the most sensitive test and is able to differentiate between different *Bartonella* species. As it was presented by Rolain, the biopsy of the lymph nodes with accompanying PCR test seems to be critical for the competent diagnosis of CSD [8].

In our study, local lymphadenopathy was observed in 17/22 children (77 %). Fifteen patients had had good reactions to the antibiotic theraphy. In two cases, the clinical course was complicated by bone inflammation.

Osteomyelitis associated with bartonellosis was described previously by many authors [15, 17, 18]. In our patients, bone lesions were usually found in association with enlargement of the lymph nodes, at the same time or after an interval of several weeks. Lymphadenopathy frequently occurred distant from the place of osteomyelitis, suggesting that bony infection could be the effect of hematogenous or lymphatic spread [18]. Clinical manifestations of bone involvement include tenderness and pain over the affected area. The radiograph images present the lytic lesions with periosteal reaction and peripheral sclerosis. Lesions could be subtle on plain radiograph, and MRI or CT should be useful to confirm characteristic abnormalities. CT presents osseous lucencies, destructive changes, and the edema of osteomyelitis. MRI is more helpful to illustrate the inflammation in bone marrow, soft tissues, and brain involvement [19].

T2-weighted, FLAIR, and T1-weighted postcontrast-enhanced sequences show increased signals in *Bartonella* osteomyelitis [20]. However, a recent case series reported two cases of multifocal bone marrow infection with *Bartonella*, with foci of increased MRI T2 signal intensity in the marrow of the sacrum, ilium, and femur, with lesions in the liver [21]. The bone biopsy reveals necrotising granulomatous inflammation.

Parotid involvement is a very rare localisation of CSD. In the histopathological examination, parotid’s tumourous lesions are caused by lymphadenitis affecting the perilymphatic tissue, resulting in swelling of the gland [22]. The fifth patient presented peripharyngeal localisation of the lesion, which is extremely rare. It made the diagnostic procedure especially difficult, because the lesion modulated the left neck vessels and left tonsil. We reviewed the literature, and there is only one pediatric case of CSD mimicking a tumour described by Yeh in 2000 [23].

Pediatricians should consider the combination of epidemiological, clinical, bacteriological, serological, and histological information, which could be evaluated in Margelith diagnostic criteria. They based on the three of four important points: 1. Cat or flea contact in spite of the presence of inoculation place; 2. Negative serological tests for the other causes of adenopathy, sterile material, and positive PCR assay from the material aspirated from a node and/or liver/spleen lesions seen on CT scan; 3. Positive enzyme immunoassay or IFA assay with a titer ratio of 1:64; 4. Biopsy showing granulomatous inflammation consistent with CSD [24].

In summary, *Bartonella henselae* infection should be considered in the differential diagnosis of children’s lymphadenopathy located in the upper extremities, head, and neck complicated by atypical masses. The combination of different methods — clinical, serological, radiological, and histological — should be the basis of correct diagnosis and treatment.
